# Antioxidant activities of some monofloral honey types produced across Minas Gerais (Brazil)

**DOI:** 10.1371/journal.pone.0262038

**Published:** 2022-01-19

**Authors:** Deosvaldo S. Pena Júnior, Clarice A. Almeida, Maria Clara F. Santos, Pedro Henrique V. Fonseca, Elytania V. Menezes, Afranio F. de Melo Junior, Murilo M. Brandão, Dario A. de Oliveira, Luciano F. de Souza, Junio C. Silva, Vanessa de A. Royo

**Affiliations:** 1 Department of General Biology, Laboratory of Natural Products, Universidade Estadual de Montes Claros, Montes Claros, MG, Brazil; 2 Department of General Biology, Laboratory of Bioprospecting and Genetic Resources, Universidade Estadual de Montes Claros, Montes Claros, MG, Brazil; 3 Cooperative of Beekeepers and Family Farmers of Northern Minas, Fazenda Bahia s/n, Bocaiuva, MG, Brazil; 4 Institute of Agricultural Sciences, Universidade Federal de Minas Gerais, Montes Claros, MG, Brazil; Bangabandhu Sheikh Mujibur Rahman Agricultural University, BANGLADESH

## Abstract

This study was carried out with the objective of determining the antioxidant properties and quantification of total phenolics and flavonoids in relation to quercetin and rutin in some of the monofloral honeys produced in Minas Gerais (Brazil). In this study, 15 monofloral honey samples were obtained from different geographic regions of Minas Gerias, Brazil. The honeys were obtained from Cooperative of Beekeepers and Family Farmers of Northern Minas. To determine the antioxidant properties of honey samples, the test methods of total phenolic content, flavonoids (rutin and quercetin) and DPPH were used. As a result of the analysis of phenolic and flavonoid contents, the samples with the best results were A1-Aroeira and A4-Assa peixe. In antioxidant activity, the honey with the best EC_50_ results was A6-Aroeira. Differences between the antioxidant activities of the honey samples were found significantly (p< 0.01).

## Introduction

Honey is a high-quality natural food product, both nutritionally and due to its therapeutic properties that ensure a balance in the biological process [1, 2] which occurs because in the composition there are bioactive compounds [3, 4].

Honey has been used for a long time in folk medicine [5, 6] orally or topically against various diseases and since then, consumers generally consider honey as a natural source of health due to the therapeutic qualities attributed to it, such as antimicrobial, gastrointestinal protective and antioxidant properties, in addition to being a good source of energy [7].

Several substances that make up honey are responsible for its antimicrobial and antioxidant character, but the chemical composition of honey is extremely variable, due to the various factors that influence the composition, such as geographic location and soil type, botanical origin, time of year, rainfall, processing, handling and storage, in addition to the bee species [8]. Thus, the nectar collected by bees for the preparation of honey, determine differences in the composition of honey. Therefore, this variation is what allows obtaining different properties, such as biological activities [9, 10].

Bioactive compounds are present in honey in minority proportions, but are responsible for these properties, such as the presence of phenolic compounds that provide those who consume honey, natural antioxidants, which help prevent disease and control aging [11–13]. The presence of phenols in honey originates from pollen and nectar collected by bees [14].

In addition to these compounds, honey has intrinsic factors, such as glucose-oxidase enzymes, catalase and other compounds such as carotenoids, organic acids, ascorbic acid, amino acids and proteins, conferring its own antioxidant activities [15].

Living cells have limited ability to nullify the activity of free radicals, but it is believed that the ingestion of antioxidants, such as those in honey, can improve cell protection and, therefore, physiological function [16]. In addition, honey activities are related to several factors, including high water and sugar content, low pH value, presence of hydrogen peroxide and other micronutrients [17–19].

The chemical and biological properties of honeys have been studied, which has brought an increased interest in the medicinal use of these honeys in treatments for diseases that are caused by oxidative, anti-inflammatory, antiviral, antifungal, antitumor stress [7, 20–21].

The content of phenolic compounds from the secondary metabolism of plants that make up honey is directly related to antimicrobial activity, those that are richer in these compounds and have a darker color usually have greater antimicrobial activity [22, 23].

Honey has been used in topical or skin applications, including the treatment of surgical wounds, skin ulcers, abscesses [24], burns and for histological preservation of skin grafts [25]. Due to their polyphenolic profile, monofloral honeys have significant antioxidant activity, as well as antidiabetic, antimicrobial and anticancer activities [26].

Manuka honey is a monofloral honey rich in macro and micronutrients, including several secondary metabolites (flavonoids, phenolic acids and 1,2-dicarbonyl compounds). Wound healing, anti-cancer, antioxidant and anti-inflammatory effects are related to the presence of these phytochemicals [26, 27].

There are honeys on the world market that have exceptional therapeutic properties and great market value, Manuka honey is an example, produced by the bee *Apis mellifera* from the nectar collected from *Leptospermum scoparium* (Myrtaceae) native to New Zealand [28, 29].

Due to the biological importance of honey in the treatment of various diseases associated with the growing need for natural alternatives, the aim is to investigate the antioxidant activity of different types of honey produced in Minas Gerais and compare the results with Manuka honey.

## Material and methods

### Chemicals and instruments

All reagents and chemicals used were analytical grade from Sigma Chemical Company (St. Louis, MO, USA). A Shimadzu UV-VIS spectrophotometer UV-VIS 2550 was used for absorbance measurements.

### Honey samples

A total of 16 samples of honey were analyzed: 15 samples from various regions of Minas Gerais (Brazil), provided by COOPEMAPI (Cooperative of Beekeepers and Family Farmers of Northern Minas, headquartered in Bocaiuva-MG) in 2019 and Manuka honey (MGO 30+) purchased from New Zealand (Brand: Manuka Health, lot: IMH 6925). The samples were identified by numbering and stored protected from light (25–30°C).

### Color determination

Performed according to the methodology proposed by the Codex Alimentarius Commission [30], which consists of reading the absorbance of the pure sample in a spectrophotometer at 560 nm against pure glycerin blank, and classification according to the Pfund table.

### Preparation of honey extracts

For the preparation of the extracts an aqueous solution of methanol 50% (v/v) was used. Then, the honey was diluted (8 mL of honey to 80 mL of 50% methanol solution). This solution was kept in a reflux device for two hours at 80°C. Dilution and extraction were performed on each honey sample separately, stored in sealed jars and kept in the freezer [31].

#### Phenolic compounds

*Determination of total phenolic content*. The Folin-Ciocalteu method was used to determine the total phenolic content [32] with some modifications. To carry out the test, the extract, at concentrations between 50 and 100% was used (600 μL) and mixed with 3.5 mL of distilled water and 200 μL of Folin-Cicalteu reagent, after 3 minutes it was added 500 μL of sodium carbonate (Na_2_CO_3_ at 3%). This solution was kept in the dark for one hour at 25°C. The absorbance was measured at 725 nm, with methanol blank, using a UV-VIS spectrophotometer (SHIMADZU-UV-VIS 2550). All measurements were taken in triplicate, and then the results were averaged and plotted on a graph of (concentration/absorbance) to determine the equation of the line and R^2^. Gallic acid (3,3,4-trihydroxybenzoic acid in concentrations between 30–80 μL/mL) was used as a standard to derive the calibration curve. The total phenolic content was expressed in mg equivalent of gallic acid per 100 g of honey [33].

*Determination of total flavonoid content*. The Aluminum chloride method was used to determine the total flavonoid content [34] with some modifications. To carry out the test, the extract, at concentrations between 50 and 100%, was used (3000 μL) and added to 200 μL of aluminum chloride (5% AlCl_3_). This solution was kept in the dark for 25 minutes at 25°C. The absorbance was measured at 417 nm, with methanol blank, using a UV-VIS spectrophotometer (SHIMADZU-UV-VIS 2550). All measurements were taken in triplicate, and then the results were averaged and plotted on a graph of (concentration/absorbance) to determine the equation of the straight line and R^2^. Rutin and quercetin (25–50 μg/mL) were used as a standard to derive the calibration curve. The total flavonoid content was expressed in mg equivalent to rutin and quercetin per 100 g of honey.

*Antioxidant activity DPPH assay (2*,*2-diphenyl-1-picrylhydrazyl)*. The DPPH radical scavenging activity of honey samples was determined as described by Brand-Williams et al. (1995) [35] with some modifications. To carry out the test, the extract, at concentrations between 50 and 100%, was used (500 μL). A DPPH stock solution of 40 μL/mL methanol was prepared. The sample was then added to 3000 μL of DPPH, shaken vigorously and kept in the dark for 25 minutes at 25°C. To obtain the standard curve of gallic acid, a stock solution at 80 μg/mL was prepared and concentrations between 30 and 80 μg/mL were used the absorbance of the solution was measured at 517 nm, using a spectrophotometer (SHIMADZU-UV-VIS 2550) against a methanol blank [35]. All measurements were taken in triplicate. With the absorbance values, the percentage of antioxidant activity was calculated by the equation:

{(AbsCont–AbsAmos) /AbsCont}×100 [36], where:
AbsCont represents the absorbance value of the control;
AbsAmos represents the absorbance value of the sample.

From the results, graphs were constructed in Excel relating concentration and percentage of Antioxidant Activity (%AA), and the EC_50_, which consists of the sample’s capacity to scavenge 50% of DPPH free radicals, was calculated through the equations of each graphic.

### Statistical analysis

The results were performed in triplicate and expressed as mean ± standard deviation. Submitted to the Shapiro-Wilk test for the normality of the ANOVA residues and verified the homogeneity between the variances. Analysis of variance (ANOVA) was applied to meet the assumptions for parametric data, with the Tukey test *a posteriori* for comparisons between means, with an alpha level of 0.05. The R open source statistical software (4.1.0) was used for analyses.

## Results and discussion

### Honey samples

The studied honeys are monofloral and are described with their respective abbreviations in Table 1.

**Table 1:** Identification of honeys according with botanical origin.

	Popular name	Scientific name	Botanical family
**A1**	Aroeira	*Astronium urundeuva* (M. Allemão) Engl.	Anacardiaceae
**A2**	Eucalipto	*Eucalyptus robusta* Sm.	Myrtaceae
**A3**	Betônica	*Hyptis sp*.	Fabaceae
**A4**	Assa peixe	*Veronia scorpioides* (Lam.)Pers.	Asteraceae
**A5**	Aroeira	*Astronium urundeuva* (M. Allemão) Engl.	Anacardiaceae
**A6**	Aroeira	*Astronium urundeuva* (M. Allemão) Engl.	Anacardiaceae
**A7**	Aroeira	*Astronium urundeuva* (M. Allemão) Engl.	Anacardiaceae
**A8**	Pequi	*Caryocar brasiliense* Cambess.	Caryocaraceae
**A9**	Candeinha	*Eremanthus incanus* (Less.) Less.	Asteraceae
**A10**	Caiaté	*Omphalea diandra* L.	Euphorbiaceae
**A11**	Aroeira	*Astronium urundeuva* (M. Allemão) Engl.	Anacardiaceae
**A12**	Cipó-uva	*Serjania lethalis* A. St.-Hil.	Sapindaceae
**A13**	Aroeira	*Astronium urundeuva* (M. Allemão) Engl.	Anacardiaceae
**A14**	Velame	*Croton urucurana* Baiil.	Euphorbiaceae
**A15**	Eucalipto	*Eucalyptus robusta* Sm.	Myrtaceae
**A16**	Manuka	*Leptospermum scoparium* J.R. Forst & G. Forst.	Myrtaceae

The honeys used in the work were obtained from the COOPEMAPI, which effectively uses the microscopic analysis of honey pollen to prove the botanical origin (S1 Table).

Honey can be categorized as monofloral and polyfloral honey. Monofloral honey is obtained from the nectar of specific source plants which can be determined by melissopalynologycal analysis. According to the International Commission for Bee Botany [37] honeys can be categorized a monofloral depending on the pollen grain size. Thus, pollen grains < 20 μm need to be at least 96% for the sample to be considered monofloral, where as pollen grains ˃ 85 μm need to be only 7% present.

### Color determination

Most of the 16 honeys evaluated showed a dark amber color (Table 2). Where 10 samples (58.82%) are characterized as dark amber, four samples (23.53%) as amber and the other three samples (17.65%) as light amber. Manuka honey has also been classified as dark amber. Color of foods is the essential factor that primarily determines the acceptability of foods [38, 39].

**Table 2:** Color shades of honeys.

Identification	Color	Identification	Color
**A1 Aroeira**	Dark amber	**A9** Candeinha	Light amber
**A2 Eucalipto**	Amber	**A10** Caiaté	Light amber
**A3 Betônica**	Amber	**A11** Aroeira	Dark amber
**A4 Assa peixe**	Dark amber	**A12** Cipó uva	Dark amber
**A5 Aroeira**	Dark amber	**A13** Aroeira	Dark amber
**A6 Aroeira**	Dark amber	**A14** Velame	Light amber
**A7 Aroeira**	Dark amber	**A15** Eucalipto	Amber
**A8 Pequi**	Amber	**A16** Manuka	Dark amber

The color of honey is related to the botanical origin, climatic factors during the flow of nectar and the temperature at which the honey matures inside the hives [40]. In addition to these factors, other factors such as mineral content also influence the color of honey. Honey color is also related to antioxidant capacity, the darker, the greater the antioxidant action, which may be linked to the presence of anthocyanin and flavone groups [41].

### Quantification of total phenolics and flavonoids

**Fig 1:**
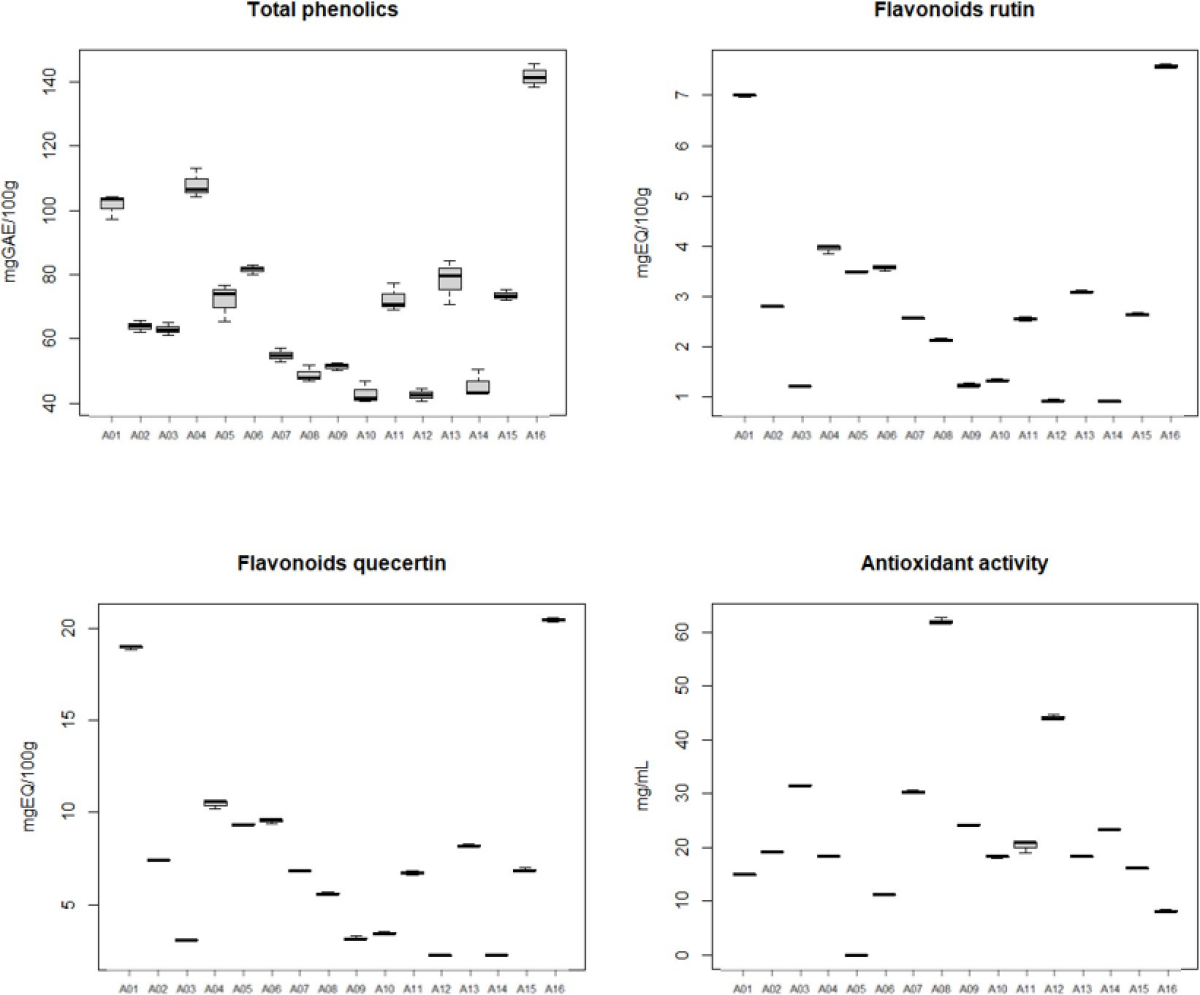
Boxplot of the comparative results obtained from honeys. For total phenolics; flavonoids equivalent to rutin and quercetin; antioxidant activity (EC_50_).

Boxplot graphical analysis was used to assess the distribution of honey performance of the 16 samples evaluated in the study (Fig 1). This analysis consists of plotting box-shaped charts where the median, the first and third quartiles of the data are represented. Box width can be used, as well as standard deviation, to assess data dispersion.

The quantification of total phenolics (Table 3; Fig 1) was made from the equation of the straight line of the standard curve for gallic acid. To calculate the equivalent value of gallic acid (mg GAE100 g^-1^ of sample), the equation of the straight line obtained (R^2^ = 0.984) was used.

**Table 3:** Data on the contents of total phenolics, flavonoids: Rutin and quercetin and EC_50_ (mean ± SD; n = 3).

	* mg/100 g of sample			
	Phenol content	Flavonoid content	Flavonoid content	EC_50_
	GAE*	Eq Rutin *	Eq Quercetin*	mg/mL
**A1**	101.67 ± 0.87^b^	7.00 ± 0.19^b^	18.94 ± 0.19^b^	15.00 ± 0.07^h^
**A2**	63.97 ± 2.33^de^	2.80 ± 0.15^f^	7.41 ± 0.13^f^	19.20 ± 0.19^fg^
**A3**	62.95 ± 2.11^de^	1.22 ± 0.15^j^	3.06 ± 0.12^j^	31.49 ± 0.17^c^
**A4**	107.93 ± 1.12^b^	3.95 ± 0.29^c^	10.47 ± 0.37^c^	18.42 ± 0.05^g^
**A5**	72.02 ± 2.00^cd^	3.49 ± 0.16^d^	9.34 ± 0.15^d^	-
**A6**	81.63 ± 2.78^c^	3.56 ± 0.20^a^	9.55 ± 0.24^d^	11.30 ± 0.05^i^
**A7**	54.91 ± 0.63^ef^	2.57 ± 0.13^g^	6.84 ± 0.12^g^	30.31 ± 0.06^d^
**A8**	48.82 ± 1.33^fg^	2.14 ± 0.17^h^	5.60 ± 0.18^h^	62.12 ± 0.13^a^
**A9**	51.38 ± 2.26^fg^	1.24 ± 0.16^ij^	3.17 ± 0.18^ij^	24.21 ± 0.09^e^
**A10**	42.93 ± 2.25^g^	1.33 ± 0.13^i^	3.45 ± 0.14^i^	18.27 ± 0.18^g^
**A11**	72.45 ± 0.17^cd^	1.90 ± 0.21^g^	4.90 ± 0.26^g^	20.30 ± 0.26^f^
**A12**	42.52 ± 3.29^g^	0.95 ± 0.20^k^	2.24 ± 0.15^k^	44.12 ± 0.15^b^
**A13**	78.29 ± 2.40^c^	3.10 ± 0.18^e^	8.19 ± 0.17^e^	18.36 ± 0.15^g^
**A14**	45.52 ± 0.52^fg^	0.92 ± 0.15^k^	2.25 ± 0.13^k^	23.33 ± 0.18^e^
**A15**	73.50 ± 3.97^cd^	2.65 ± 0.23^g^	6.89 ± 0.23^g^	16.15 ± 0.11^h^
**A16**	141.73 ± 2.37^a^	7.58 ± 0.26^a^	20.43 ± 0.26^a^	8.20 ± 0.16^j^

Antioxidant activity tests were performed in triplicate, three times, in sample 5; and in all tests the results showed non-relevant antioxidant activity. EC_50_ = 0.269 mg/mL of gallic acid. Means followed by the same letter in the column do not differ according to Tukey’s test at p ≤ 0.05.

To obtain the total flavonoid content (Table 3; Fig 1), two standards were used, rutin and quercetin, and the values were calculated in the same way as described above. For rutin the equation of the line with R^2^ = 0.998, for the quercetin with R^2^ = 0.999.

The determination of the total phenolic content is considered a quick and sensitive method for honey samples [42]. In the vast majority of results, it is possible to observe appositive relationship between the result of total phenolics and the levels of flavonoids, with the antioxidant capacity in honeys [43].

The content of phenolic compounds ranged from 42.52 ± 3.29 to 107.93 ± 1.12 milligrams equivalent to gallic acid per hundred gram of honey (mg GAE100 g^-1^), where the most relevant results were for A4-Assa-peixe (107.93 ± 1.12 mg GAE100 g^-1^), A1-Aroeira (101.67 ± 0.87 mg GAE100 g^-1^) and A6-Aroeira (81.63 ± 2.78 mg GAE100 g^-1^) respectively, when compared to gallic acid and also when compared to Manuka honey (A16) (Table 3).

Even the lowest values found for honeys in this work, such as A8-Pequi (48.82 ± 1.33 mg GAE100 g^-1^); A14-Velame (45.52 ± 0.52 mg GAE100 g^-1^); A10-Caiaté (42.93 ± 2.25 mg GAE100 g^-1^) and A12-Cipó uva (42.52 ± 3.29 mg GAE100 g^-1^), are equal to or superior to Brazilian monofloral and multifloral honeys from the Southeast region with contents between 18 and 43 mg GAE100 g^-1^ [44]. Intermediate levels observed in this study (62.72–81.63 mg GAE 100 g^-1^), has higher contents than honeys analyzed in Malaysia with a content of 59.05 mg GAE100 g^-1^ [45]; Cuban honeys with a content of 59.58 mg GAE 100 g^-1^ [46] and with Portuguese honeys, the highest value observed was 72.78 mg GAE 100 g^-1^ [47]. The results of the levels for honeys A1-Aroeira and A4-Assa peixe (101.67 ± 0.87 and 107.93 ± 1.12 mg GAE100 g^-1^) has levels close to the values verified for multifloral honey from different geographical origins, with a higher value of phenolic compounds from 110 mg GAE 100 g^-1^ [48] and also honeys of Spanish origin with a maximum observed content of 103.48 mg GAE100 g^-1^ [49].

When comparing the values of 15 honeys to Manuka honey (141.73 ± 2.37 mg GAE 100 g^-1^) used in this study as a positive control, it can be observed that A4-Assa peixe honey (107.93 ± 1.12 mg GAE100 g^-1^) is the closest to the total phenolic content.

The flavonoid content in the analyzed honey samples ranged from 2.24 ± 0.15 to 18.94 ± 0.19 mg QE 100 g^-1^of honey. The highest contents were of honeys A1-Aroeira, A4-Assa peixe, A6-Aroeira and A5-Aroeira, respectively (Table 3). When comparing the contents with other studies, it can be observed that the content determined for A1-Aroeira of 18.94 ± 0.19 mg QE 100 g^-1^ is higher than that evaluated for multifloral honeys from Argentina, where the flavonoid content is 15 mg QE100 g^-1^ [50]. A study with multifloral Spanish honeys presented, for flavonoids, 2.39 mg QE100 g^-1^ [49], which is close to the lowest values found in this study, which are for A12-Cipó uva and A14-Velame honeys (2.24 ± 0.15 and 2.25 ± 0.13 mg QE 100 g^-1^) respectively.

The flavonoid contents ranged from 0.92 ± 0.15 to 7.00 ± 0.19 milligrams equivalent to rutin per gram of honey (mg RE 100 g^-1^) were also found. The highest value was determined for the A1-Aroeira sample and the other samples with intermediate results have contents between 2.14 ± 0.17 and 3.95 ± 0.29 mg RE 100 g^-1^

When comparing the flavonoid values with Manuka honey (20.43 ± 0.26 mg QE 100 g^-1^ and 7.58 ± 0.26 mg RE 100 g^-1^), it is observed that the sample A1-Aroeira is that the contents related to quercetin and rutin (18.94 ± 0.19 mg QE 100 g^-1^ and 7.00 ± 0.19 mg RE 100 g^-1^), are closest to the results observed for Manuka honey.

### Antioxidant activity

One of the methods used to assess the antioxidant potential of extracts is the colorimetric assay based on the capture of the DPPH radical (2,2-diphenyl-1-picryl-hydrazyl), which is characterized by an oxidation-reduction reaction, where the DPPH radical is reduced in DPPH-H, identified by an initially purple to yellow color change, as well as a decrease in absorbance at 515/517 nm [51].

To determine EC_50_ in mg/mL (Table 3; Fig 1) for the antioxidant action, the equations of the standard lines for gallic acid and for each of the honeys were used. The R^2^ of the equations were between 0.995 and 0.919.

Among the antioxidant compounds of natural origin, the phenolic compounds stand out, which have defense functions against pest attacks, but in the human body they have oxide-reduction properties, which can play an important role in the absorption, sequestration and neutralization of free radicals, in addition to inhibiting lipid peroxidation [37].

The EC_50_ result observed for cipó-uva honey (44.12 mg/mL) and wild honey (19.20 mg/mL) are higher than those found in a study carried out in Piauí for cipó-uva honey (237.70 mg/mL) and wild honey (136.92 mg/mL) [52].

After analyzing the results, it was found that samples A6-Aroeira and A1-Aroeira had the best EC_50_ result, respectively 11.30 mg/mL and 15.00 mg/mL, with the A6 sample being the closest of the result obtained for Manuka honey (A16 = 8.20 mg/mL) (Table 3). It is important to emphasize that the lower the EC_50_ value, the greater the efficiency of the sample in deactivating the free radical.

The presence of aromatic rings and hydroxyls in phenolic compounds, which gives this class the antioxidant power and thus, are responsible for the antioxidant, antimicrobial, antiviral and antitumor activities of honey, which contributes to human health in preventing diseases [53].

Antioxidants also have the function of preventing free radicals from damaging cells and tissues [53]. The main classes of antioxidants that are present in nature are phenolic compounds (phenolic acids, flavonoids and tannins), carotenoids, tocopherols, ascorbic acid and its derivatives [54–60].

The detection of phenolic compounds and the ability to scavenge free radicals by the studied honeys allow us to infer that honeys present themselves as a functional food, since through their antioxidant activity, interact with free radicals, fighting them, and thus help in various cellular processes, such as the prevention of skin aging [61].

## Conclusion

The studied honeys have levels of total phenolics and flavonoids above the results found in the literature for other honeys, mainly A1-Aroeira and A4-Assa peixe honeys. Regarding the evaluation of the antioxidant action, EC_50_ have promising results, especially the A6-Aroeirahoney sample. Honeys with the same flowering, but from different geographic regions, have different values of chemical composition, further studies are needed to verify the differences in results between these honeys. The continuity of studies in relation to monofloral honeys is necessary to determine other physicochemical parameters. But the results presented in this work are promising to guide other works to be carried out with monofloral honeys, mainly related to studies of biological activities.

## Supporting information

S1 Table Honey analysis information provided by COOPEMAPI [1–4]. (PDF)
