# Supplementary material for: Antioxidant activities of some monofloral honey types produced across Minas Gerais (Brazil)
Source: PLoS One. 2022 Jan 19;17(1):e0262038. doi: 10.1371/journal.pone.0262038 (PMC8769325; doi:10.1371/journal.pone.0262038)
Supplement: S1 Table — (PDF) [file pone.0262038.s001.pdf]

Table S1. Honey analysis information provided by COOPEMAPI [1, 2, 3, 4].

	Pollen type	Pollen count	Index %
**A1**	*Astronium urundeuva*	600	98.52
	Croton urucurana	5	0.82
	Malvastrum sp	2	0.33
	Citrus sinensis	1	0.16
	Baccharis calvescens	1	0.16
**A2**	*Eucalyptus robusta*	300	75
	Astronium urundeuva	100	25
**A3**	*Hyptis sp*	213	69.38
	Croton urucurana	30	9.77
	Eucalyptus robusta	28	9.12
	Baccharis calvescens	11	3.58
	Astronium urundeuva	10	3.26
	Mimosa scabrella	4	1.30
	Protium sp	3	0.97
	Sida sp	2	0.65
	Serjania lethalis	2	0.65
	Cecropia glazioui	2	0.65
	Anadenanthera colubrina	2	0.65
**A4**	*Veronia scorpioides*	500	100
**A5**	*Astronium urundeuva*	200	79.36

	Eucalyptus robusta	50	19.84
	Coffea arábica	2	0.79
**A6**	*Astronium urundeuva*	200	94.79
	Eucalyptus robusta	10	4.74
	Struthanthus SP	1	0.47
**A7**	*Astronium urundeuva*	500	83.19
	Piptadenia communis	100	16.64
	Croton urucuana	1	0.16
**A8**	*Caryocar brasiliense*	200	98.52
	Serjania lethalis	3	1.48
**A9**	*Eremanthus incanus*	20	66.66
	Hyptis sp	7	23.33
	Unidentified	3	10
**A10**	*Omphalea diandra*	500	100
**A11**	*Astronium urundeuva*	200	95.69
	Eucalyptus robusta	4	1.91
	Schizolobium parahyba	3	1.44
	Croton urucurana	2	0.96
**A12**	*Serjania lethalis*	182	66.66
	Astronium urundeuva	64	23.44

	Hyptis umbrosa	19	6.95
	Eucalyptus robusta	4	1.40
	Baccharis calvescens	2	0.73
	Protium SP	2	0.73
**A13**	*Astronium urundeuva*	400	96.15
	Eucalyptus robusta	13	3.13
	Cambessedesia hilariana	2	0.48
	Serjania lethalis	1	0.24
**A14**	*Croton urucurana*	150	83.34
	Anadenanthera colubrina	10	5.55
	Eucalyptus robusta	20	11.11
**A15**	*Eucalyptus robusta*	100	95,24
	Serjania lethalis	5	4,76

Data provided by COOPEMAPI. Analyzes are performed following the references: [1] AOAC - Association of Official Analytical Chemists. Official methods of analysis. Washington DC. 1998; [2] Barth, O.M. Melissopalinology in Brazil: A review of pollen analysis of honeys, propolis and pollen loads beens. Agricultural Science 2004; 61(3):342-350; [3] Codex Alimentarius. Food and Agriculture Organization of the United Nations (FAO). Codex Standard for Honey. 2001; [4] Louveaux, J., Maurizio, A., & Vorwohl, G. Methods of melissopalynology. Bee World, 1978; 59(4):139-157.
